# Tiger-Striped PASH: Recognition of a Unique Morphology Allows for a Zippered-Up Diagnosis of Pseudoangiomatous Stromal Hyperplasia of Breast

**DOI:** 10.1155/2021/7697987

**Published:** 2021-12-26

**Authors:** Mohamad Sakibuzzaman, Ryan W. Kendziora, Toshi Ghosh, Malvika H. Solanki, Amy Lynn Conners, Lisa J. Ahlberg, Charles D. Sturgis

**Affiliations:** ^1^Department of Laboratory Medicine and Pathology, Mayo Clinic, Rochester, MN, USA; ^2^Department of Radiology, Mayo Clinic, Rochester, MN, USA; ^3^Department of Internal Medicine, Mayo Clinic, Rochester, MN, USA

## Abstract

Pseudoangiomatous stromal hyperplasia (PASH) of the breast is histologically characterized by anastomosing and slit-like spaces invested by collagenous stroma and lined by flattened, spindle cells. These clear spaces that may mimic microscopic vascular channels do not contain red blood cells. Immunohistochemistry (IHC) studies may also help to confirm a diagnosis of PASH, with the spindled cells marking positively with CD34 and PR while demonstrating no reactivity with more specific endothelial antigens such as CD31 and ERG. In the current case, a 39-year-old female was diagnosed with cellular PASH of the right breast with unique histological patterns showing “tiger-striped” and “zippered” histologies. To our knowledge, this is the first report of these unique variant PASH morphologies.

## 1. Introduction

Pseudoangiomatous stromal hyperplasia (PASH) is a relatively infrequent, benign, proliferative breast condition [1, 2]. PASH is a spindle-cell proliferation that pathologically originates from collagen-producing myofibroblasts [3]. Initially, Vuitch et al. described nine cases of PASH in 1986 [4]. The existing literature has reported approximately 1,500 cases to date [5]. Although the exact trigger for development of PASH remains controversial and needs to be clarified, hormonal contributions to myofibroblastic proliferation have been suggested as likely causes [3]. Knowing this, it is not unusual that PASH is most commonly reported in hormonally active premenopausal women. Some authors have suggested that hormone replacement therapy in postmenopausal women may be a potential risk factor for PASH [6]. Specific clinical and radiological diagnoses of PASH are challenging, as patients with PASH may be labeled with other diagnoses such as fibroadenoma, fibrocystic changes, and others by physical exam and radiologic studies [1, 3, 7]. In general, histological examination is required to establish an accurate diagnosis.

While classical PASH is most often readily and accurately identified by breast pathologists, some variant morphologies such as cellular PASH may be challenging to histologically diagnose and may be confused with other spindle cell entities such as fibromatoses, fasciitis, myofibroblastomas, phyllodes tumors, nerve sheath tumors, low-grade angiosarcomas, and others [3]. It is, therefore, important to recognize variant light microscopic morphologies of PASH and to be familiar with ancillary testing which may prove useful in delineating cell types and classifying lesions with morphological overlap. Currently, there are no published definitive treatment guidelines for PASH. Routine surgical interventions are not supported by the American Society of Breast Surgeons. Clinical surveillance is likely to be a key management strategy for most patients, as no association with malignancy or propensity to progress to malignancy is known to exist [3]. In this report, we describe a case of PASH with unique histological features including “tiger-striped” and “zippered” morphologies.

## 2. Case Presentation

**Figure 1:**
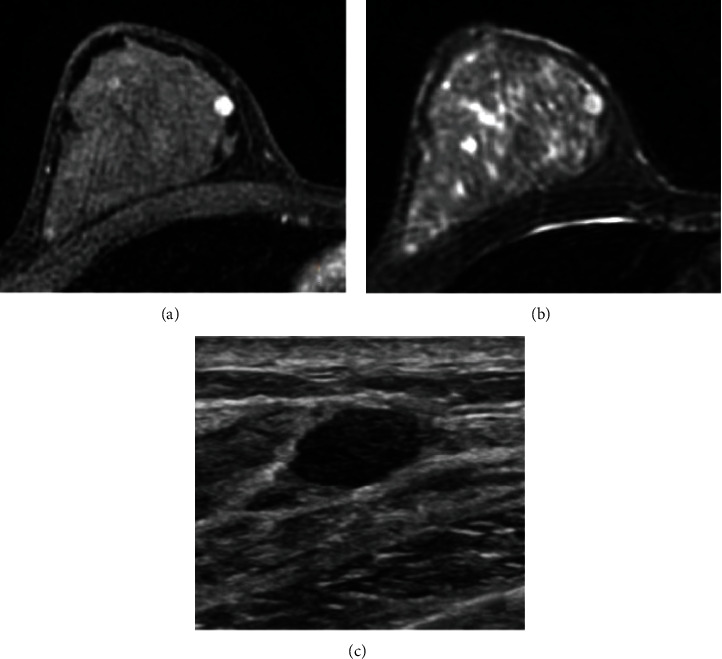
(a) MRI of the right breast with IV gadolinium shows an oval enhancing mass in the upper inner right breast middle depth; (b) T2-weighted images show bright signal, though less bright than cysts seen elsewhere in the breast. Slit-like spaces are not definitely appreciated, possibly due to the small size of the finding. (c) Second-look ultrasound shows an oval, microlobulated, hypoechoic mass in the upper inner right breast, corresponding with that seen on MRI.

**Figure 2:**
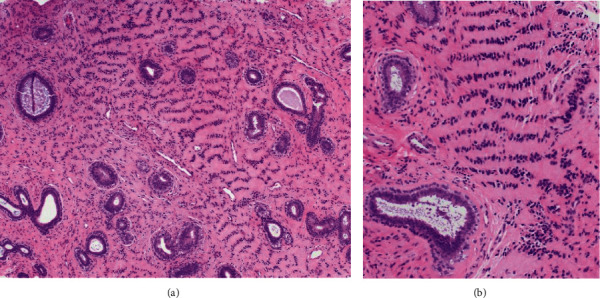
Low-power histologic assessment shows scattered nonneoplastic breast epithelial elements including scattered ducts and the edge of a terminal duct lobular unit with stromal expansion between and within these epithelial structures. Eosinophilic expanses of fibrous tissue are noted with linear arrangements of spindle cell nuclei resulting in a tiger-stripe-like pattern ((a) hematoxylin and eosin, 100X). At intermediate magnification, the nuclear arrangements with offset eosinophilic cytoplasmic bands emphasize the striped morphology ((b) hematoxylin and eosin, 200X).

**Figure 3:**
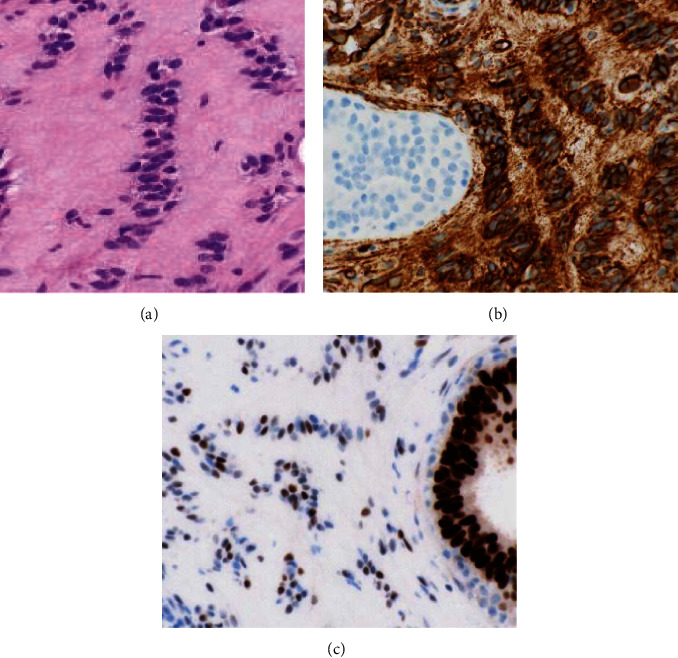
High-power histologic assessment confirming a zippered morphology with interlacing lesional nuclei oriented perpendicular to the cleft space ((a) hematoxylin and eosin, 400X). The lesional spindled cells demonstrate diffuse and strong immunoreactivity with CD34 while the adjacent epithelium is nonreactive ((b) CD34 IHC, 600X). The lesional spindled cells also show immunoreactivity with progesterone receptor ((c) PR IHC, 600X).

A 39-year-old white, non-Hispanic female presented with a painless palpable left breast mass after self-examination. The palpable mass had been stable without change in size over several months. She was G1P1, having been pregnant at age 32, and breastfed for 1 year. The patient had a family history of breast carcinoma with her mother being diagnosed with breast cancer at the age of 29. She had a history of premenopausal menorrhagia and was taking norgestimate and ethinyl estradiol. She also had a history of polycystic renal disease with no significant interval changes. Physical examination revealed a 1.5 × 1.0 cm painless soft mobile mass located in the superior medial quadrant at the 11 o'clock position. There was no associated lymphadenopathy. Her most recent mammogram, approximately three months remote, was negative. Because of her family history and palpable mass, bilateral breast MRI was recommended. Breast MRI revealed no abnormal findings in the left breast, but did show an oval, enhancing mass in the upper inner right breast. Subsequent ultrasound showed an isoechoic mass in the left breast, which corresponded to the palpable abnormality and was felt compatible with lipoma. The right breast ultrasound showed a 0.8 × 0.7 × 0.6 cm oval circumscribed mass which corresponded with that seen in MRI (Figure 1). Biopsy was recommended. The ultrasound-guided core biopsy specimen showed a population of lesional spindle cells with groups of palisaded nuclei. These cells exhibited an interlobular distribution but also surrounded and invested ducts and lobules. The cells were cytologically bland with no significant nuclear atypia or pleomorphism (Figure 2). The morphologic differential diagnosis included cellular PASH, myofibroblastoma, schwannoma, and perhaps others. Immunohistochemistry (IHC) studies were performed. These confirmed the spindled cells to be immunoreactive with CD34 (diffusely strongly positive), SMA (diffusely moderatley positive), PR (variably moderately positive), ER (focally weakly positive), and RB (no loss of RB) (Figure 3). The spindled cells were nonreactive (negative) with desmin, S100, SOX10, and P63.

The combined histomorphology and IHC results confirmed a cellular variant of PASH with a unique “tiger-striped” or “zippered” morphology (Figures 2–3). Since the PASH was an incidental imaging finding in the breast, no further intervention was recommended, and the patient was advised to continue with annual imaging and monthly breast self-examination. The imaging findings were felt compatible with the pathologic diagnosis of PASH. As the pathologic fiindings were benign, the patient did not undergo genetic testing.

## 3. Discussion

PASH is a rare benign breast disorder that originates from the reactive proliferation of stromal myofibroblasts. It is most commonly encountered as an incidental histologic finding during the evaluation of benign or malignant breast lesions [1, 6–10]. Less frequently, it can present as a palpable mass, which is called nodular PASH or tumorous PASH [6, 9, 10]. Multifocal nodules or diffuse lesions with asymmetric enlargement of the breasts have also been reported on rare occasions [7, 9]. Clinically, nodular PASH appears as a well-circumscribed, firm, mobile mass without any changes in the overlying skin and can mimic fibroadenoma [6, 7]. Although PASH is most commonly found in premenopausal women [1–3, 6–8, 10], it can affect patients from different age groups ranging from 12 to 75 years [6, 10]. It can also affect men with gynecomastia [1, 3, 6, 7, 10, 11]. PASH has been reported in immunocompromised patients, including patients with HIV and neurofibromatosis type 1, and patients undergoing cyclosporine treatment [7, 10].

The exact pathogenesis of PASH is still not well defined, but hormone-dependent proliferative changes in the stroma are widely accepted causal factors [1, 3, 6–8, 11, 12]. A hormonal hypothesis is further substantiated by the increased propensity of PASH to occur in premenopausal women and in postmenopausal women taking hormone replacement therapy [3, 6–8, 11]. Progesterone has been suggested to play a key role in the pathogenesis of PASH. It causes aberrant proliferation of stromal myofibroblasts. Increased expression of progesterone receptors has been demonstrated in myofibroblast nuclei by immunohistochemical staining [3, 6–8]. Estrogen is felt to perhaps play a less significant role in the pathogenesis of PASH, and lesser degrees of estrogen receptor positivity have been described in PASH by IHC [6].

Radiological studies such as mammography and ultrasonography can provide preliminary diagnostic information, but confirmation via histopathology is necessary to reach a final diagnosis. In mammography, nodular PASH appears as an oval or round, noncalcified, well-defined hyperdense lesion [6–8]. In ultrasonography, PASH is most frequently described as a homogenous, well-circumscribed, hypoechoic mass [1, 6–8]. MRI may reveal an isointense mass on T1-weighted images, and linear reticular lacelike patterns are observed in T2-weighted gradient-echo images [6]. Though PASH may appear as a mass, it most commonly appears as nonmass enhancement with persistent kinetics [12]. Following a suspicious imaging finding, a core needle biopsy is sufficient to confirm the diagnosis in nearly all patients [3]. Findings in PASH fine-needle aspiration cytology are nonspecific, but needle aspiration samples may be useful in ruling out other differential diagnoses, such as malignant lesions or fibroepithelial lesions [6].

Grossly, nodular PASH is identified as an unencapsulated, well-defined, round to oval, rubbery mass with gray, white homogenous cut surfaces. Except for lesion that have previously undergone fine-needle aspiration or core biopsy, necrosis and hemorrhage are usually absent [6–8, 13]. The classic histological finding of PASH is networks of anastomosing slit-like pseudovascular spaces interspersed within a dense hyalinized stroma. These slit-like spaces are lined by spindle cells that contain attenuated nuclei and lack mitotic activity and atypia [1, 3, 5–8, 10, 13]. The slit-like spaces are devoid of intrinsic erythrocytes.

On IHC, myofibroblasts in PASH are positive for CD34, vimentin, and SMA (smooth muscle actin) and are negative for cytokeratin, S100, and endothelial markers such as factor VIII, CD31, and ERG [5–9]. Nuclei of stromal myofibroblasts also frequently stain positive for progesterone [6, 8].

Fibroadenomas can be confused with nodular PASH because they share similar clinical and radiological characteristics [3, 6, 7, 14]. Fibroadenomas are in most instances easily distinguished from PASH on microscopic examination. The characteristic findings of fibroadenoma are glandular elements dispersed in intralobular stroma. The glandular elements may show either pericanalicular or intracanalicular patterns of growth [6]. Other common differential diagnoses include low-grade angiosarcoma, phyllodes tumor, myofibroblastoma, and schwannoma [6–10]. Angiosarcoma is a rare malignant breast tumor of vascular origin that marks positively for CD31, CD34, factor VIII, and ERG [8, 9, 14]. Like PASH, myofibroblastoma also originates from an aberrant proliferation of myofibroblasts and demonstrates similar features such as fascicular foci and positivity for CD34, vimentin, and SMA [6, 8]. However, PASH is often immunoreactive for progesterone receptors and shows typical slit-like spaces, which are not anticipated in myofibroblastomas [6]. Myofibroblastomas are generally immunoreactive with desmin, while PASH is not. The characteristic features of phyllodes tumor are hypercellular stroma forming leaf-like structures, containing cleft-like spaces that are lined by epithelial cells [8]. Another differential diagnosis might be schwannoma, which is much less frequently reported in the breasts than PASH. Histologically, schwannomas show Antoni A and Antoni B regions. Antoni A regions contain highly cellular components arranged in compact and palisaded patterns, while Antoni B regions have myxoid components with less cellularity. Schwannian tumors may also show Verocay bodies with nuclear palisading [15, 16]. Nerve sheath tumors are composed of spindle cells having relatively uniform nuclei with fascicular arrangements. The spindle cells in schwannomas strongly express S-100.

Management of PASH is nonuniform, and standardized guidelines do not exist [3]. Surgical excision and periodic surveillance are the available options, and choices for treatment depend upon the specifics of individual clinical presentations [7, 8]. Tumorous or nodular PASH is most commonly managed with surgical excision [6–8]. Sometimes, wide excision of breast tissue or mastectomy with reconstruction is required to manage diffuse lesions, those lesions with persistent pain and recurrent tumors [6, 7]. Incompletely excised PASH can recur in up to 22% of cases and can be managed with reexcision [6]. No additional interventions are required for incidentally diagnosed PASH in concordant core biopsies [1, 2, 7]. Sufficient data on the efficacy of medical management is unavailable; as until now, only anecdotal case reports have demonstrated the possible efficacy of antihormonal therapy in preventing PASH progression [6, 11]. PASH is not a premalignant condition and does not increase the risk of malignancy [6–9, 17]. It has an excellent prognosis, and no PASH-related deaths has been reported [8, 17].

## 4. Conclusion

PASH is considered a benign lesion of the breast. There have been many studies on PASH describing its clinical, radiological, and histological properties since the publication of Vuitch et al.'s original article. The histological features of PASH are described as anastomosing slit-like spaces lined by single flat cells in dense fibrous perilobular or intralobular stroma. In this paper, we present a case where the spindle cell proliferation was present in both interlobular and intralobular distributions. The lesional cells exhibited a unique “tiger-striped” low to intermediate power histoarchitecture. On high magnification, the lesional cells took on a “zippered” morphology with tapered nuclei appearing to interlace at right angles to the orientation of stromal clefts. By reporting this unique PASH morphology, we hope to mitigate diagnostic dilemmas and assist in proper identification and patient management.

## Data Availability

All data comes from Mayo Clinic pathology and radiology.
